# Raising awareness of suicide prevention during the COVID‐19 pandemic

**DOI:** 10.1002/npr2.12141

**Published:** 2020-10-06

**Authors:** Jianyu Que, Kai Yuan, Yimiao Gong, Shiqiu Meng, Yanping Bao, Lin Lu

**Affiliations:** ^1^ Peking University Sixth Hospital Peking University Institute of Mental Health NHC Key Laboratory of Mental Health (Peking University) National Clinical Research Center for Mental Disorders (Peking University Sixth Hospital) Beijing China; ^2^ National Institute on Drug Dependence Peking University Beijing China

**Keywords:** COVID‐19, infectious disease, suicide prevention

## Abstract

Suicide is one of the top 20 leading causes of death worldwide. With the rapid spreading of coronavirus disease 2019 (COVID‐19) crisis around the world, suicide cases induced by the COVID‐19 pandemic have been reported in many countries. Individuals with suspected and confirmed COVID‐19 infection, frontline healthcare workers, bereaved families, elders, children, and adolescents are vulnerable populations who might be at elevated suicide risk. In this micro‐review, a systematic search through PubMed was performed for a comprehensive investigation of suicide risk factors during the pandemic. On this basis, we put forward considerations and advice for preventing pandemic related suicide, including staying socially connected through online platform or apps during period of quarantine, reducing unemployment, dispelling rumors and misinformation in time, and maintaining evidenced‐based management of psychiatric symptoms. More importantly, early detection and timely intervention of individuals with psychiatric disorders and suicide behaviors will be effective to reduce the number of suicides, with specific measurements of using validated scales to perform regular suicide risk screening, improving the availability of mental health services, and providing appropriate and evidence‐based interventions for individuals in demand. Policy makers, psychiatrists, psychologists, and other healthcare professionals need to collaborate to control the possible suicide events during the COVID‐19 pandemic and future possible crisis.

## Abstract 2

Individuals who are exposed to bio‐disaster might encounter increased suicide risk. Investigation of infectious disease‐related risk factors would facilitate suicide prevention. Early detection and timely intervention can be key and effective way to reduce suicide cases.

## Graphical abstract

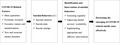

Suicide accounts for approximately 800 000 deaths worldwide annually, which is a serious global public health issue that urgently needs to be addressed.^1^ Identifying factors that are associated with a higher risk of suicide is valuable for suicide prevention. Early detection and timely intervention of individuals with suicidal behaviors is crucial for reducing suicide cases. The coronavirus disease 2019 (COVID‐19) pandemic, one type of bio‐disasters, is a tremendously traumatic event that may increase suicide risk.^2^, ^3^ Managing negative effects of bio‐disaster with timely and proper interventions is of paramount importance.

The COVID‐19 pandemic has been spreading dramatically worldwide, putting people in distress due to high risk of infection and quarantine. The pandemic not only harms physical and mental health of the public but also has a major adverse economic impact within a short period of time because of disruptions in employment, daily transportation, global industrial and supply chains, and international trade. Such adverse consequences, in turn, can cause tremendous psychosocial stress which may increase the possibility of suicide. During the COVID‐19 crisis, suicide cases have been reported in many countries, including United States,^4^ Italy,^5^ Germany,^5^ England,^6^ and India.^7^ Most of the suicide victims were frontline healthcare workers and individuals with suspected or confirmed COVID‐19 infection. In the early period of COVID‐19 pandemic, a top emergency room doctor at Manhattan Hospital and the Finance Minister of Germany committed suicide while participating in the anti‐pandemic work,^4^, ^5^ thereby eliciting people's concern about suicide risk and prevention during this crisis. Individuals were not only under unprecedented psychological pressure but also forced to suffer stigma and discrimination when being directly exposed to COVID‐19 virus or bereaved family in this bio‐disaster, associated with a higher suicide risk.^8^, ^9^, ^10^ Besides, many studies indicated that children, adolescents, and older adults were the vulnerable populations that might be associated with a higher risk of suicide during the pandemic of infectious disease.^11^, ^12^, ^13^, ^14^, ^15^, ^16^, ^17^ Hence, recognizing that the burden produced by psychological impacts of COVID‐19 may exceed which by the pandemic itself is in urgent need.^18^

In this micro‐review, search strategy of “(Suicid*) AND (Infectious Disease[Title/Abstract] OR Epidemic[Title/Abstract] OR Communicable Disease[Title/Abstract] OR COVID‐19[Title/Abstract])” was used to identify relevant studies from PubMed on July 17, 2020. A total of 454 records were yielded after initial search, and 64 relevant articles that investigating the relationship between infectious diseases and suicide risk were reserved for reading carefully. Then, we, as much as possible, tried to investigate the infectious disease‐related factors for increased suicide risk, and on this basis, providing some considerations and advice to prevent the development of suicide during the COVID‐19 pandemic. Early detection and effective intervention of individuals with suicide behaviors is crucial to reduce suicide cases.

First, keeping physical distancing was an important measure to hinder the spread of COVID‐19, which was implemented and shown effective in many countries. However, we need to recognize the large negative impact of these anti‐epidemic measures on economic recession and public health, especially among children and adolescents.^19^ Recently, some studies have reported suicide cases triggered by the adverse effects of mass quarantine, and staying at home could increase suicide risk through thwarted belongingness.^20^, ^21^, ^22^, ^23^ It is noteworthy that physical distancing measures themselves were shown not associated with elevated incidence of suicidal behaviors,^24^ which indicates that mass quarantine related suicide was probably because of social disconnection. Therefore, keeping socially connected through online platform or apps may help prevent suicide during the pandemic of COVID‐19.^24^

Second, economic recession induced by COVID‐19 itself and anti‐epidemic measures has commonly occurred. Worsening economic conditions and increasement of unemployment was another pandemic related factor that has been proven associated with a higher risk of suicide.^22^, ^25^, ^26^, ^27^, ^28^ Nordt et al^29^ have conducted an estimated model to explore the association between suicide and unemployment and found that suicide risk associated with unemployment was elevated by 20%–30%. Therefore, governmental financial assistance and debt relief policies are urgently needed to lower unemployment rate and ensure basic daily needs for families in economic crisis.^28^, ^30^

Third, positive and objective information about COVID‐19 needs to be widely disseminated to the public. Social network services have been flooded with negative information and misinformation during this extraordinary time for certain political purposes, which can result in mental health problems for high‐risk groups and vulnerable individuals.^31^ Moreover, media reports of people committing suicide may be followed by copycat suicides, especially when details of the method of suicide are specified.^32^ To minimize the negative impacts of rumors and misinformation, mass media, healthcare organization, clinicians, researchers and scientists must work together to conduct health education to disseminate accurate information about the COVID‐19 pandemic and dispel the rumors and misinformation in time, and for rumor mongers, relevant laws and regulations should be strictly implemented.^33^ These measures could make rumors and misinformation under control, and consequently abate suicide risk.

Fourth, association between suicide and mental disorders (ie, depression, bipolar disorder, insomnia, and drug addiction) is well established. Anti‐pandemic measures, such as mass quarantine, transportation restrictions, and city lockdown, during the COVID‐19 pandemic increased possibility of interrupting psychiatric patients' maintenance treatment and making more substance addicts to suffer from withdrawal symptoms, which may increase suicide risk of these populations in this special period.^34^, ^35^, ^36^ Besides, sleep disturbance is an independent risk factor for suicidality, and a recent study has found that insomnia severity fully accounted for the positive association between COVID‐19‐related fear and suicidal ideation.^37^ Treating insomnia by evidence‐based interventions, such as cognitive behavior therapy for insomnia, would facilitate suicide prevention. Managing psychiatric symptoms and maintaining treatment of psychiatric patients is crucial for reducing suicide risk.

Fifth, a history of suicide attempts is the strongest predictor of subsequent suicide, identification, and intervention of suicide behavior as early as possible was shown conducive to suicide prevention.^38^ Using widely validated and uncomplex scales, such as the Columbia Suicide Severity Rating Scale, to perform regular screening for vulnerable populations was an effective means for early detection of suicide risk.^38^ In addition, an individualized psychiatric evaluation would be further conducted by mental health professionals when the screenings were positive. Although screening tools have promising application foreground, the evidence to support routine use of suicide screenings in emergency departments and in primary care is weak.^39^ One possible reason was that 45%–76% individuals who committed suicide sought help from primary care providers who were not ready to cope.^40^ Combining with objective markers including brain imaging and gene polymorphisms, establishment of a more clinically valued suicide risk prediction model to regularly screen vulnerable populations and follow‐up may effectively reduce COVID‐19 related suicide cases. Equally important is the need to add referral systems as an adjunct to managing individuals with suicidal behavior.

Sixth, improving the availability of mental health services for people during COVID‐19 is necessary. Chinese government has successively set up more than 600 free counseling hotlines to help with mitigating individuals' psychological stress and fear of being infected, including eleven 24‐hour hotlines that provide free psychological services for healthcare workers.^41^ Many psychiatric hospitals established platforms to provide online mental services for existing patients with diagnosed mental disorders, in order to lessen the rate of self‐reduced or self‐stopped medication. Additionally, 415 mental health professionals from other provinces went to Hubei province to provide face‐to‐face interventions for high‐risk individuals.^41^ Easier access to mental health services can also effectively prevent suicide.

Seventh, appropriate and effective interventions as well as strong social support and mobilization are needed to reduce suicide risk. COVID‐19 infection is a traumatic life event that can make people feel hopeless and heighten suicide risk, hence, it is necessary to help infected individuals build up faith and confidence to fight against the disease. Besides, increased access to lethal means was associated with a heightened risk for suicide. For extremely high risk individuals, restricting access to means of suicide including decreasing access to pesticides and reducing availability of firearm are highly recommended.^38^, ^42^ Government and community support as well as adequate access to personal protective equipment and medical supplies are vital to protect individuals from succumbing to fear and worry, thereby lessening the risk of suicide that is associated with this pandemic. Moreover, appropriate personalized interventions are also needed.

Exposure to the COVID‐19 pandemic is associated with individuals' increased risk of suicide. Making efforts from whole society to minimize the negative impact of infectious disease‐related risk factors would facilitate suicide prevention. Early detecting suicide behaviors and providing proper and timely interventions are vital to prevent the tragedy of suicide in various high‐risk groups. Suicide is preventable, with early identification of the predictors for suicide and treating it with evidence‐based measures. Policy makers, psychiatrists, psychologists, and other healthcare professionals need to be attentive to possible suicide cases during the COVID‐19 pandemic and prevent the long‐term consequences of COVID‐19 pandemic on elevated suicide risk.

## CONFLICT OF INTEREST

The authors declare no conflict of interest.

## AUTHOR CONTRIBUTIONS

JQ and LL proposed the topic and the main idea. JQ and YG collected related literature material. JQ wrote the initial draft. KY, YG, SM, YB, and LL revised manuscript with critical content. YB made the final revision. All authors contributed to the final draft.

## Data Availability

Data sharing not applicable to this article as no datasets were generated or analyzed during the current study.
